# What’s in a commercial meal kit? Structured review of Australian meal kits

**DOI:** 10.1017/S1368980023000265

**Published:** 2023-06

**Authors:** Fiona H McKay

**Affiliations:** The School of Health and Social Development, Institute for Health Transformation, Deakin University, Locked Bag 20,00, Geelong, VIC 3220, Australia

**Keywords:** Meal kit, Nutrition content, Food price, Food preparation

## Abstract

**Objective:**

The aim of this project is to review the products and recipes contained within popular subscription meal kits to determine if they are suitable for wider use among people who are food insecure.

**Design:**

Across the 6-week period, weekly meal kits from both HelloFresh and Markey Spoon were purchased, resulting in thirty-six individual meals that were prepared and assessed. Meals were assessed based on the content included in the meal kit compared with the recipe card and the nutrition panel, the costs of the individual foods if purchased at one of two major supermarkets and the ease of preparation.

**Setting:**

Australia.

**Participants:**

Households were comprised of two, 2-person households who were provided with 2 meals each week, and two, single-person households who were provided with one meal each week.

**Results:**

The findings of this research suggest that while the meal kits are convenient and, in general, the recipes are easy to follow, and the meals would be made again, the high levels of salt and fat may preclude these kits from regular inclusion in a healthy diet. The meal kits were also found to be more costly than the same ingredients if purchased from a major supermarket. However, the convenience of having most of the foods needed to prepare a full meal with little to no wastage may counterbalance this cost.

**Conclusions:**

Meal kits may be a useful component of a healthy diet, that can increase meals prepared and consumed in the home, and thanks to the clear instructions and pre-portioned ingredients, may reduce stress related to food preparation.

The challenges related to adopting a healthy diet, high rates of obesity^(1)^, diabetes^(2)^, diet-related non-communicable diseases^(3)^ and poor diet quality that includes an insufficient consumption of fruit and vegetables^(4)^ have led some policymakers and researchers to promote home cooking as a way to improve eating habits^(5,6)^. Positive benefits of eating at home include lower odds of having high cholesterol, being overweight and having excess body weight^(7)^. Cooking at home has been identified as an important health behaviour for both low- and high-income households^(6)^, has been associated with higher food security^(6,8)^ and has long been recognised as a health-promoting activity^(5,9)^. There has been a large amount of research exploring the impact of cooking at home on diet, with this research suggesting that frequently cooking meals at home is associated with consuming lower energy^(10,11)^, lower sugar and fat consumption^(10)^, greater consumption of fruits and vegetables^(5)^, better diet quality^(10,12)^, psychological benefits^(13)^ and reduced eating out or purchasing takeaway foods^(14,15)^.

People are cooking at home less now than in the past. The possible reasons for this have been widely explored and are said to include the cost of living pressures, a decline in cooking skills, a lack of knowledge and confidence in the kitchen, chances in household income, time, home infrastructure and enjoyment and satisfaction with cooking^(14,16–21)^. Both diet quality and cooking frequency are related to income or socio-economic status^(22–24)^. While there is evidence suggesting that cooking at home has positive benefits for health, the association between cooking meals at home and diet quality is complex^(25)^. There are also important cultural considerations and familiar routines that need to be taken into account when describing and encouraging home cooking^(26,27)^.

Despite these barriers, meals cooked at home are generally found to be healthier than meals sourced elsewhere. A study from the United Kingdom found that eating more home-cooked meals was associated with better diet quality including increased fruit and vegetable and micronutrient intake,^(7)^ and a study from the USA found home cooking was associated with increased intake of fibre and decreased energy and sugar consumption^(10)^, while other studies have suggested that home cooking results in improved diet quality and cooking knowledge and confidence, as well as positive psychological outcomes^(28,29)^. There is also a financial benefit to cooking at home. Several studies have demonstrated the cost saving of cooking at home^(11)^, with and without the consideration of associated time costs^(21)^.

One lesson from the COVID-19 pandemic is the increase in accessibility, popularity and variety of foods available for home delivery and the changes that people made to their eating habits during long periods of home isolation^(20,30,31)^. Even before the pandemic, meal kits like HelloFresh and Marley Spoon were increasing in popularity^(32)^; however, their use significantly increased during the pandemic^(19,33,34)^. While meal kits are marketed as convenient, there remain questions surrounding the nutritional value of popular meal kits. Several studies have sought to answer these questions. For example, a recent study compared the nutritional qualities of twelve recipes (3 per week for 4 weeks) from five different meal kits available to Australian consumers^(35)^. The findings of this study suggest that meals were high in fat and Na and low in fibre. Moores et al^(36)^ extended this research by exploring the qualities of recipes available online within meal kits to understand their nutritional benefits or risks. They explored the nutritional composition of HelloFresh recipes available online over a 12-month period, finding that while meals contained vegetables and had some health-promoting properties, some were very high in Na and may not be suitable for regular consumption or as a part of a healthy diet. These findings suggest that interventions that include meal kit could be useful for people who are food insecure and/or who are reliant on emergency and community food assistance. The wide availability and choice within the market for meal kits mean that they are readily available, and the simple instructions may allow an opportunity to improve confidence and skills^(37)^. This current research seeks to extend what is already known by comprehensively reviewing meal kits from two main companies over a 6-week period, including a review of contents, nutritional composition, costs and ease of use. This research will provide evidence for the feasibility and acceptability of these products and may be considered useful for work with hungry and food-insecure populations.

## Method

This study employed several methods to determine the acceptability, feasibility and nutritional composition of meal kits. This study sought to provide pilot evidence for the usability of subscription meal kits for a range of households. Ethical approval was granted by the Deakin University Human Ethics Committee (HEAG-H 12_2022).

### Data collection

There are an increasing number of subscription meal kits available to Australian consumers. Consumer advocacy group Choice identified five commonly available meal kit services: HelloFresh, Marley Spoon, Pepper Leaf, Dinnerly and Everyplate^(38)^. The two meal kits that were the most highly rated in the Choice review are also those with the largest market share in Australia^(39,40)^, are easily available to most Australian geographies and as such were chosen to be included in this research. For the purpose of this study, subscriptions to both HelloFresh and Marley Spoon meal kits were taken for a 6-week period. Each service provides a range of options as a part of their subscription: including the number of people the meal kit is to provide food for (2–4 people), the number of recipes included in each box (3–5 recipes) and the type of recipes that the box should include (vegetarian, family-friendly, low calorie, quick). To ensure comparability across meal kits, meal kits from both services were purchased catered for two people, with three recipes, with the automatically generated selection of meals (classic menu). All meal kits were delivered to the author each week. After the contents were recorded (see below), individual complete meal kits were delivered to the four households who agreed to be involved. Households were known to the author and comprised of two, two-person households who were provided with two meals each week and two, single-person households who were provided with one meal each week. There were three main components under investigation as a part of this study.

#### Nutritional composition of the meal kits

Data extraction included documentation of the actual foods provided to determine if there were any discrepancies against the advertised content, the number and type of ingredients provided as well as additional ingredients required at home and total cooking and preparation time. Pantry staples that were included as a recipe item, but not included in the meal kit, were also recorded; when preparing the meals, participants were asked to follow these quantities to report any deviation from the recipe.

#### Cost of the meal kits

The individual costs of the foods provided in the meal kit (excluding any discounts) were determined by comparing the items provided to the cost of purchasing the same foods in the same (or similar) quantities from the two main supermarkets (Coles and Woolworths). This information was used to determine the cost per meal if purchased from the supermarket.

#### Recipe acceptability and preparation

All meals provided in the meal kits were prepared by one of four households who agreed to be included in the project. After preparing the meal, participants were asked to answer ten questions (through an online Qualtrics survey, see supplementary material) to determine how easy the recipe was to follow, how easy the meal was to cook and if it fits their expectations. All meals were prepared according to the recipe, and any changes were documented.

### Data analysis

Analysis of the nutritional information was conducted via the FoodWorks software version 10 (AusFoods, AusBrands nutritional composition databases) with the actual food provided in the meal kit to determine the accuracy of the information provided in the nutrition panel. Nutritional data provided per serving as reported on the nutrition information panels on recipe cards were extracted and included energy (kJ), protein (g), total fat (g), total carbohydrates (g), from both HelloFresh and Marley Spoon and saturated fat (g), sugar (g) and Na (mg) from HelloFresh only. Consistent with the work of Gibson and Partridge^(35)^, this information was then used to determine the nutritional value of the meals against the 30 % of the daily nutrient references values Australian Dietary Guidelines, as Australians typically eat three meals/d, one meal should provide around one-third of a person’s daily nutritional requirements. Where there are differences between recommended intakes between men and women, the recommendation for males aged 31–50 years was chosen.

Data extracted were macronutrient, vitamin and mineral composition. Data were exported from Foodworks into Excel 365 for analysis. Continuous data are presented as means and standard deviations, and categorical data are presented as *n* (%).

A cost analysis of the meal kits was conducted. Ingredient costs were estimated by searching online supermarket webpages (Coles online and Woolworths online) to identify the same (or similar) quantities of available ingredients. The results of the cost analysis were compared against the costs of the meal kits (excluding discounting).

Descriptive statistics were used to summarise the results of the menu preparation component of this study. Question sought to determine the feasibility and ease of the meal instructions with photographs used to determine if the recipe could be easily prepared and presented the same as the instructions.

## Results

Across the 6-week period, weekly meal kits from both HelloFresh and Markey Spoon were purchased, resulting in thirty-six individual meals that were prepared and assessed. Meals were assessed based on the content included in the meal kit compared to the recipe card, the costs of the individual foods if purchased at one of two major supermarkets and the ease of preparing the meals.

### Nutritional composition of the meal kits

The actual ingredients of the meal kits were documented and entered into FoodWorks version 10 for analysis, a summary of the results of which is presented in Table 1 – see supplementary material for breakdown based on each meal. On average over the 6-week period, the meals from HelloFresh were higher in energy, protein, fats and carbohydrates than those of Marley Spoon. There were also more serves of grains and vegetables in the HelloFresh kits. The HelloFresh kits had, on average, twice the amount of sodium as the Marley Spoon kits (1256 g *v*. 741 g) both values which are higher than 30 % of the Nutrient Reference Value from the Australian guidelines, while other minerals were similar. Dietary levels of Ca were not achieved by either meal kit while Mg was not achieved by Marley Spoon. Vitamins were estimated to be similar in both meal kits, expect for Vitamin A which was higher in the HelloFresh kits (1840 µg *v*. 980 µg).

**Table 1:** Nutrition analysis of meal kits (30 % NRV)

Macronutrients	Total NRV (30 %)		HelloFresh (actual)		Marley spoon (actual)	
	*n*	%	Mean	sd	Mean	sd
Energy (kJ)	–		3676	696	2678	760
Protein (g)	64	19·2‖	48	6	44	9
Total fat (g)	–		46	13	29	19
Saturated fat (g)	–		15	7	8	4
Polyunsaturated (g)	–		6	3	5	5
Monounsaturated (g)	–		21	8	12	10
Carbohydrate (g)	–		64	25	46	23
Sugars (g)	–		17	8	12	6
Dietary fibre (g)	30	10	11	4	10	3
Minerals						
Na (mg)	2000	600**	1256	568	741	590
K (mg)	3800	1140	1577	366	1472	343
Mg (mg)	420	126	131	28	120	19
Ca (mg)	1000	300	265	110	191	85
P (mg)	1000	300	614	103	582	115
Zn (mg)	14	4·2	5	2	5	2
Fe (mg)	8	2·4	5	2	5	2
Vitamins						
Thiamine (mg)	1·2	0·36‖	0·6	0·4	0·6	0·4
Riboflavin (mg)	1·3	0·39‖	0·5	0·2	0·5	0·2
Niacin (mg)*	16	4·8‖	22·8	8·6	22·1	8·7
Vitamin C (mg)	45	13·5‖	66·0	54·4	80·5	33·0
Vitamin E (mg)	10	3¶	8·4	5·6	7·1	4·2
Vitamin B_6_ (mg)†	1·3	0·39‖	1·1	0·7	1·2	0·6
Vitamin B_12_ (µg)	2·4	0·72‖	1·8	1·6	1·4	0·8
Vitamin A (µg)‡	900	300‖	1840	1574	980	871
Food groups (No. of serves)§						
Grains	–		2·01	1·64	1·76	1·67
Refined	**–**		2·01	1·64	1·70	1·71
Vegetable	–		3·85	2·09	3·2	1·78
Dark green vegetables	–		0·35	0·61	0·36	0·44
Red and orange vegetables	–		2·14	2·49	0·68	0·78
Starchy	–		0·41	0·68	0·57	0·88
Legumes	–		0·03	0·11	0·01	0·05
Other veg	–		0·92	0·76	1·58	1·01
Protein	–		1·6	0·45	1·58	0·26
Dairy	–		0·34	0·30	0·1	0·22

NRV, Nutrient Reference Value.

* Niacin equivalent.

† By analysis.

‡ Total vitamin A equivalents.

§ The FoodWorks software provides analysis based on serves of food groups based on the Australian Guide to Healthy Eating, please see https://xyris.com.au/ for more detail.

‖ Recommended dietary intake.

¶ Adequate intake.

** Suggested dietary target.

When considering the average macronutrient components across the 6-week period, meal kits from HelloFresh had a larger percentage of energy from fat (46 %) than then kits from Marley Spoon (38 %). This likely accounts for the higher energy value of the HelloFresh kits. Marley Spoon had a larger percentage of energy from protein (23 % *v*. 30 %), and other macronutrients were similar (see Table 2).

**Table 2:** Macronutrients as a percentage of total energy

	HelloFresh	Marley spoon
Protein	23	30
Total fat	46	38
Carbohydrate	29	29
Fibre	2	3
Other	1	1

**Table 3:** Provided *v*. actual nutrition information, per serve

	HelloFresh (provided)		HelloFresh (actual)		Marley spoon (provided)		Marley spoon (actual)	
	Mean	sd	Mean	sd	Mean	sd	Mean	sd
Energy (kJ)	3546	525	3676	696	2904	447	2678	760
Protein (g)	48	6	48	6	42	7	44	9
Total fat (g)	43	14	46	13	33	9	29	19
Saturated fat (g)	15	7	15	7	–		8	4
Carbohydrate (g)	68	20	64	25	53	21	46	23
Sugars (g)	17	6	17	8	–		12	6
Na (mg)	1514	504	1256	568	–		741	590

Both HelloFresh and Marley Spoon provide some nutritional information for each recipe. The provided information was compared against the actual food provided. On average, the information provided by HelloFresh was similar to that calculated, except for Na which was estimated to be lower than the information provided. Marley Spoon provided less information than HelloFresh, only providing information related to total energy, protein, total fat and total carbohydrate. All Marley spoon calculations were similar to the provided information (see Table 3).

### Cost of meal kits

Food costs were estimated for each recipe for the two major supermarkets (see Table 4). Individual recipe costs were calculated by determining the price of the required portion of each ingredient for each recipe. In addition to the ingredients supplied, each meal kit assumed that the household would contain ‘pantry staples’ which across the 6-week period included: olive oil (both extra virgin and regular), butter, Dijon mustard, soy sauce, milk, sugar, vinegar (both white and red wine), honey, flour, eggs and salt and pepper. This represents a one-off cost of $37·85 if these products were purchased from Coles and $38·85 if purchased from Woolworths.

**Table 4:** Cost per recipe

	Coles	Woolworths	Purchase price
Week 1			
Marley spoon			
Chicken Piccata	$13·41	$11·33	
Miso Salmon	$12·89	$13·72	
Pulled pork tacos	$23·26	$21·35	
Total	$49·56	$46·40	$78·99
HelloFresh			
Bacon and mushroom spaghetti	$7·13	$7·88	
Beef and veggies	$8·89	$10·53	
Chicken and creamy peppercorn sauce	$9·47	$12·00	
Total	$25·49	$30·41	$75·93
Week 2			
Marley spoon			
Greek beef pockets	$19·64	$19·87	
Laksa	$16·30	$14·04	
Garlic pepper pork steaks	$13·19	$11·87	
Total	$49·13	$45·78	$78·99
HelloFresh			
Beef cottage pie	$14·75	$13·72	
Chorizo and spinach risotto	$15·56	$16·35	
Miso chicken Japanese salad	$15·82	$10·85	
Total	$46·13	$40·92	$75·93
Week 3			
Marley spoon			
Beef rump sandwich	$22·63	$23·05	
Chicken pot pie	$10·71	$8·19	
Ginger-Hoisin Lamb and Greens	$15·22	$16·68	
Total	$48·57	$47·92	$78·99
HelloFresh			
Cheesy spiced pork burger	$10·94	$8·26	
Crumbed chicken dippers	$19·05	$15·04	
Korean style beef tacos	$15·67	$14·09	
Total	$45·66	$37·39	$75·93
Week 4			
Marley spoon			
Chargrilled beef rump	$21·41	$21·65	
Chimichurri-Crusted chicken pork steak	$13·50	$12·69	
Spicy Asian Chicken Burger	$15·42	$10·51	
Total	$50·33	$44·85	$78·99
HelloFresh			
Chermoula beef meatballs	$14·31	$12·78	
Easy Sweet Chilli pork	$11·46	$17·28	
Spiced chicken and cheesy sweet potato mash	$13·24	$10·36	
Total	$39·01	$40·42	$75·93
Week 5			
Marley spoon			
Chicken noodles	$14·87	$14·21	
Golden seared chicken	$14·98	$14·21	
Tex mex salmon	$14·91	$19·76	
Total	$44·77	$48·19	$78·99
HelloFresh			
Aussie spiced chicken schnitzel	$14·77	$12·44	
Chorizo and Tomato Orecchiette	$17·46	$17·06	
Indian beef keema curry	$14·40	$14·50	
Total	$46·63	$44·00	$75·93
Week 6			
Marley spoon			
Baked chicken with tomato and pumpkin	$12·94	$14·44	
Beef steak	$14·78	$14·89	
Beef steaks	$19·11	$18·74	
Total	$46·83	$48·07	$78·99
HelloFresh			
Pork and red pesto meatballs	$9·92	$9·57	
Sweet chilli chicken burger	$15·24	$11·64	
Beef and spinach pie	$11·25	$12·74	
Total	$36·41	$33·95	$75·93

Food and costs per recipe ranged from the lowest cost recipe, Bacon and Mushroom Spaghetti, HelloFresh, priced at $7·13 from Coles and $7·88 from Woolworths; to the highest cost recipe, Beef Rump Sandwich, Marley Spoon, priced at $22·63 from Coles and $23·05 from Woolworths. Across the whole study period, the average costs for the food included in the weekly meal kits would have been less if purchased at either supermarket (see Table 5). The weekly cost of the Marley Spoon boxes over the 6-week period (excluding discounts) was $78·99, the same food from Coles was $48·20 and $46·87 from Woolworths, suggesting an average of over $30 convivence fee each week. The weekly cost of the HelloFresh boxes over the 6-week period (excluding discounts) was $75·93, and the same food from Coles was $39·89 and $37·85 from Woolworths, similarly suggesting an average of over $30 convivence fee each week.

**Table 5:** Cost per meal box

	Coles	Woolworths	Meal kit purchase price	Coles difference	Woolworths difference
Week 1					
Marley spoon	$49·56	$46·40	$78·99	$ 29·43	$ 32·59
HelloFresh	$25·49	$30·41	$75·93	$ 50·44	$ 45·52
Week 2					
Marley spoon	$49·13	$45·78	$78·99	$ 29·86	$ 33·21
HelloFresh	$46·13	$40·92	$75·93	$ 29·80	$ 35·01
Week 3					
Marley spoon	$48·57	$47·92	$78·99	$ 30·42	$ 31·07
HelloFresh	$45·66	$37·39	$75·93	$ 30·27	$ 38·54
Week 4					
Marley spoon	$50·33	$44·85	$78·99	$ 28·66	$ 34·14
HelloFresh	$39·01	$40·42	$75·93	$ 36·92	$ 35·51
Week 5					
Marley spoon	$44·77	$48·19	$78·99	$34·22	$30·80
HelloFresh	$46·63	$44·00	$75·93	$29·30	$31·93
Week 6					
Marley spoon	$46·83	$48·07	$78·99	$32·16	$30·92
HelloFresh	$36·41	$33·95	$75·93	$39·52	$41·98
Total average difference				$33·42	$35·10
Total averages					
Marley spoon	$48·20	$46·87	$78·99	$ 33·78	$ 35·33
HelloFresh	$39·89	$37·85	$75·93	$ 32·23	$ 34·38

### Recipe acceptability and preparation

Over the 6-week period, thirty-six meals were prepared, eighteen each from Marley Spoon and HelloFresh. Of the thirty-six recipes, almost all (*n* 35, 97 %) were considered very easy, or easy to follow, with just recipe one somewhat difficult to understand, twenty-three (64 %) of the recipes would be made again by participants. The size of most meals (*n* 26, 72 %) was considered ‘just right’, while nine (25 %) were considered to be too big and just one was considered to be too small. Most meal preparation resulted in no or limited food wastage (*n* 26, 72 %). Meals took on average 41 min to prepare (range 20–90 min, sd 14·8). This was greater than the suggested preparation and cooking time described on the recipe (33 min, range 20–55 min, sd 8·4). All meals required some pantry staple. This included oil (*n* 23, 64 %), vinegar (red, white, or balsamic) (*n* 13, 36 %), butter (*n* 8, 22 %), sugar (8, 22 %), mustard (*n* 7, 19 %), soy sauce (*n* 7, 19 %) and eggs (*n* 5, 14 %). Kitchens were also expected to be stocked with a range of cooking utensils, oven, stove and a range of pans and pots. Most recipes (*n* 21, 58·3 %) were made without any changes. Those that were altered were done so because of taste preferences (for example leaving out olives), because the cooker did not have a ‘pantry staple’ (for example, Dijon mustard) or to create more food (for example, including an additional potato to make a larger serve of mashed potato), no participants reported adding additional table salt.

## Discussion

This research sought to review meal kits from two main companies over a 6-week period. This review included an analysis of the contents and nutritional composition of the meals in the kits, a comparison of the costs of the kits and the cost of the foods if purchased from a major supermarket and ease of use of the recipes. The findings of this research suggest that while the meal kits are convenient and, in general, the recipes are easy to follow and the meals would be made again, the high levels of salt and fat may preclude these kits from regular inclusion in a healthy diet. The meal kits were also found to be more costly than the same ingredients if purchased from a major supermarket. However, the convenience of having most of the foods needed to prepare a full meal with little to no wastage may counterbalance this cost.

The findings of this study are consistent with other published research on meal kits. Previous research suggests that meal kit interventions can be logistically feasible, utilised and acceptable to participants in a range of settings^(37,41,42)^. For example, in a pilot study exploring feasibility, acceptability and outcomes, including skills, confidence and intake. Horning^(37)^ found that participants reported increased confidence when cooking, as well as improved cooking techniques, and the availability of food suggests that interventions that include a meal kit component may have the potential increasing healthy, home-cooked meals. Likewise, Utter *et al*.^(13,41)^ conducted two studies to test the feasibility and acceptability of providing meal kits to families in New Zealand as a way to increase the number of home-cooked meals and overcome barriers of time. These studies found that by providing families with meal plans, including recipes and ingredients, meals were prepared at home and families ate together, with families identifying a range of benefits for their families. While a study from the USA found that while families are interested in cooking and eating together, they face barriers of cost and time that could be overcome through the provision of meal kits^(43)^.

Consistent with other studies that explored the nutritional content of meal kits, this study found that meal kits on average were high in Na. Analysis of Australian dietary data suggests that Na from the evening meal represents approximately one-third of daily Na intake^(44)^. The suggested dietary target of sodium for Australian adults is 2000 mg/d (or 600 mg/evening meal). On average for both Marly Spoon and HelloFresh meals, this daily target was exceeded. This finding is consistent with the work of Moores^(36)^ and Gibson and Partridge^(35)^, who identified Na content in meal kits as well above the suggested daily targets, with the implication that regular use of meal kit services may lead to an increase in Na intake and the associated health impacts.

While these meal kits are easy to use and convenient, possibly one missed opportunity is the chance to increase nutrition and cooking literacy. These meal kits are comprised of pre-portioned ingredients separated into kits for each of the meals for the week and include step-by-step recipe cards with photographs. However, the portions are larger, possibly as suggested by Moores^(36)^ to reflect value for money, and recipe instructions contain a number of assumptions about the resources and level of skill of the person preparing the meals, often excluding steps that may be assumed knowledge (for example, tipping out water used for boiling potatoes before mashing), but that for someone without basic cooking skills may not be present. In addition, the generic or house labelling of some of the foods (for example HelloFresh Garlic and Herb seasoning) make the recipes difficult to recreate without the meal kit. For the purpose of nutrition and cooking education, if these kits included common ingredients with supermarket packaging or noted alternatives, then households may be able to remake the meal on a subsequent occasion.

Despite these mixed findings, interventions that seek to address food insecurity or assist people who are reliant on emergency and community food assistance to prepare healthier meals may benefit from the inclusion of meal kits. Previous research has found that providing meal kits to households with adolescents increased home-cooked meals and decreased household food insecurity^(13,41)^. While another study that bundled ingredients and recipes for clients of emergency and community food assistance found that the practice increased the selection of healthy foods compared to individuals making their own food choices^(45)^.

### Limitations

While there are clear findings from this study, there are several limitations that need to be taken into consideration. This small size of the sample, thirty-six recipes over 6 weeks with only two meal kit companies, mean that there are seasonal changes in the products that have not been included here. However, the length is consistent with other similar research and serves the purpose for which this research was designed, namely, to review contents, nutritional value, costs and ease of use of meal kits. This research was also conducted in a home setting, not in a lab setting. This may mean that the FoodWorks analysis is imprecise; however, the real-world setting provides a realistic overview of the feasibility of preparing these meals. Participants who were engaged to prepare and review the meals were known to the author and are generally healthy and of high income and cooking skill. This may mean that they possess higher skills and resources than other groups, particularly those who may experience food insecurity or who have chronic health conditions. When considering the cost data, it needs to be considered that it is often more cost-effective to purchase foods in bulk. This was not taken into account with the closest quantity of food selected from the supermarket for comparison. As such, definitive claims cannot be made about the cost differences between meal kits and the same meals from supermarkets other than to suggest that the same food is cheaper from supermarkets.

## Conclusions

The results of this study suggest that the meal kits may be a useful component of a healthy diet that can increase meals prepared and consumed in the home, and thanks to the clear instructions and pre-portioned ingredients, may reduce stress related to food preparation. The results of this study add to the meal kit literature by analysing actual food provided by meal kit companies against their nutritional panel, by cooking the meals to determine ease of process and by comparing the costs of the meals to the same or similar goods from a major supermarket.

## Supplementary material

For supplementary material accompanying this paper visit https://doi.org/10.1017/S1368980023000265. click here to view supplementary material
