# Supplementary material for: What’s in a commercial meal kit? Structured review of Australian meal kits
Source: Public Health Nutr. 2023 Feb 9;26(6):1284–92. doi: 10.1017/S1368980023000265 (PMC10346045; doi:10.1017/S1368980023000265)
Supplement: Supplementary file 1 [file S1368980023000265sup.zip › S1368980023000265sup001.docx]

1. What recipe did you make tonight?
2. How long did it take to make?
3. Did you have everything that you needed provided?
   1. If no, what did you need to provide from your own supply?
4. Was the recipe easy to understand?
5. Would you make this meal again?
6. Was there anything that was not suited to your taste?
   1. Too salty
   2. Not salty enough
   3. Too spicy
   4. Not spicey enough
   5. Too fatty
   6. A bit bland
7. Did you make any modifications to the recipe?
8. How was the portion size?
   1. Too big
   2. Too small
   3. Just right
9. Up load a photo of the final meal and any other photos that you took while preparing the meal
